# Medico-Chirurgical Transactions. Vol. XXIII. [Concluding Notice]

**Published:** 1841-04-01

**Authors:** 


### Medico-Chirurgical Review,

### No. LXVIII.

**[No. 28 of a Decennial Series.]**

JANUARY 1, to APRIL 1, 1841.

Medico-chirurgical Transactions, Published by the Royal Medical and Chirurgical Society of London. Vol. XXIII. London: Longman and Co. 1840.

[Concluding Notice.]

We resume our examination of the contents of this volume, with a—

Case of large Osseous Tumor of the Uterus. By James M. Arnott, Esq. Surgeon to the Middlesex Hospital.

A maiden lady, aged 72, was run against by a big dog, and thrown forwards on the pavement, on the 18th of February, 1840. There was a large tumor in the abdomen, and upon this the lower part of the ileum being struck, was burst. She died of fæcal extravasation and peritonitis in thirty-four hours.

On removing, says Mr. Arnott, the tumor, which was effected with some difficulty, so firmly was it impacted in the upper opening and cavity of the pelvis, the bladder was found attached to it in front, low down; but the uterus could not be readily made out. However, on tracing the vagina upwards, the cavity of the uterus was discovered in the shape of an elongated, very narrow canal, stretching along the posterior surface of the tumor, over which the Fallopian tubes were likewise spread out. The *form* of the uterus had *entirely*, and its *substance*, in a great measure, disappeared; for while its posterior parietes, forming the back part of the elongated canal, were reduced to a state of extreme atrophy, so as to resemble membrane, the *anterior* had become expanded and stretched over the surface of the tumor, which had clearly been originally developed in its substance, and was now covered throughout by a very thin, but more or less distinct, layer of uterine tissue.

The tumor was of an irregular oval shape, being larger at the upper end. It measured *seven* inches in length, *nineteen* in circumference in the direction of the oval, *fourteen* round at the distance of an inch from its upper end, *thirteen* at the same distance from its lower. The colour was yellowish white: the surface slightly tuberculated or botryoidal. It weighed, as has been already stated, five pounds. On being sawn through, it was found as hard as marble, and quite solid: yet the section presented an appearance as if the mass had been formed of several separate portions firmly agglomerated; an *appearance* arising, however, from minute traces of fibrous tissue being here and there still perceptible in it. Attached to the upper extremity of the large one, but distinct from it, there were several small tumors, varying in size from that of a pea to a chestnut, and which presented precisely the same structure."

Professor Daniell found that it had the following composition.

| | |
|---|---|
| Animal matter, including water and ammoniacal salts.......... | 35· |
| Phosphate of lime, with a small quantity of phosphate of magnesia, | 56· |
| Carbonate of lime...................................... | 5. |
| Alkaline sulphates, phosphates, and muriates ................. | 4· |
| | 100· |

It turned out that the lady had discovered the tumor, when of the size of a goose's egg, in 1808. She consulted Dr. Denman, who privately informed her friends that it was cancer of the uterus in an inactive state.

The case is interesting, and is communicated in Mr. Arnott's usual perspicuous style.

### II. ON THE RAPID ORGANIZATION OF LYMPH IN CACHEXIA. By JOHN DALRYMPLE, Esq. Assistant Surgeon to the Ophthalmic Infirmary, Moorfields.

Mr. Dalrymple, the author of an excellent treatise on Diseases of the Eye, and an accomplished ophthalmic surgeon, labours, in this paper, to establish the conclusion that organization of plastic effusions is more facile in the cachectic than in the robust.

Those, he says, who have been accustomed to witness ophthalmic diseases on a large scale, cannot fail to be at once struck with the greater tendency to the effusion and organization of fibrine on the surface of the iris in syphilitic cases than in those of idiopathic iritis, and there will be no difficulty in admitting that the specific cases occur, at least in this metropolis, in by far the greater proportion, in enfeebled constitutions;—in those debilitated by excesses, irregularity of moral habits, or the mal-administration of mercury in the primary disease.

The first appearance of the tubercles of fibrine, which is the form such deposits generally assume, is observed at the annulus minor of the iris, where the capillary vessels are most numerously interlaced, more minute, and constitute the extreme circulation of the part. Gradually, however, as the affection extends, we find these deposits occupying various spots of the anterior plane of the membrane, while from a yellowish colour they assume a red hue, more or less distinctly marked, manifesting the organization of the fibrine.

"Again," he continues, "in those affections of the posterior part of the eye in children, which we have been accustomed to call malignant, almost every one has been surprised to observe, in the early stage, how healthy appear to be the subjects of this frightful malady. During the period of the existence of the bright and metallic reflexion from the deposit at the posterior part of the globe, the child appears to retain its ordinary health, although perhaps the disease may be going on to increase. But so soon as you begin to trace the organization of the fibrine, indicated by the red points, or ramifications of actual vessels, not only may you expect a great deterioration of the general health, but also the rapid progress of the affection.

It is then curious to inquire whether the decline of health precedes, accompanies, or follows this organization, or rather, whether it is the cause or consequence. I do not hesitate to declare my belief, that it is the cause rather than the effect; first, because I have often seen the disease existing many months prior to the organization of the deposit, and upon an accidental impairment of health it has suddenly assumed an active form, and gone on unchecked to a fatal termination; and secondly, because, when in a particular case, the health of the child had by great care been much improved, the disease itself appeared to have been checked, and the globe began to waste; and lastly, because in this same case, when the negligence of the parents again allowed the health of the child to decline, the disease burst forth with redoubled activity, and went on rapidly to a fatal termination." 209.

From these considerations, he turns to injections of morbid parts. These he thinks prove the same ready organization of the materials of the blood, in enfeebled conditions of the system.

Mr. Dalrymple first describes the injected preparation of a knee-joint amputated for extensive disease. He refers more particularly to the abscesses in the cellular tissue and amongst the ligaments.

These particular cysts were of various sizes; all, however, more or less small, some containg pus, others a cheesy and almost concrete material. All the cavities, however, were lined with what at first appeared to be unorganized fibrine, varying from half a line to two lines in thickness, seldom smooth, but rather rugous, and on its free surface somewhat nodulated or granular. This fibrine was detached with so much facility from the vascular membrane lining the abscesses, as to lead one to conclude it was of recent origin, and had no vascular communications with the highly-injected membrane, from which it was evident it had been poured out. When, however, some portions of this fibrine had been carefully removed, dried on plates of glass, and submitted to the microscope, it immediately became evident that it was not only organized, but minutely traversed with vessels, injected, *ex votis*, with coloured size.

When a portion of fibrine had been removed in adhesion with the vascular, or what is often called the pyogenic membrane, the continuity of the vessels from the latter into it was delicately exhibited. If a portion of both was cut in profile, or if the fibrine was carefully reversed from the surface of the pyogenic membrane, the same appearances were exhibited. Again, when a portion of the fibrine adhering to the vascular web of the abscess was viewed from its free surface, the penetrating power of the instrument showed these delicate capillaries shooting up from below and ramifying to the surface of the effusion.

In a case of scurvy, under the care of Mr. Busk, of the Dreadnought hospital ship, a coagulum appeared to have become organized.

" A seaman was received on board the Dreadnought in the last stage of sea scurvy, of which he shortly died. One of the legs of this man was injected, and on examination it was found that a large deposit of coagulated blood adhered to the bone of the tibia, covered by periosteum, which was raised and separated from the bone from the tuberosity nearly to the ancle. On each of the three sides of the tibia was the same appearance observed. The clots measured from three to four inches in length, and in thickness from a quarter to half an inch. The periosteum which firmly adhered to the blood, was divided and reflected above and below, so as to expose the external surface of the clot. The injection had surprisingly succeeded, and the dark coagula were seen, even with the naked eye, studded in every part with red points, as of the torn mouths of vessels that had entered from the periosteum.

Sections of this blood, made perpendicularly to the bone, exposed numerous branches all filled with coloured size, and which appeared at first sight as if they were the vessels that had originally passed directly from the periosteum to the outer lamella of the tibia, but raised and elongated by the separation of the membrane, and traversing the surrounding effused coagula.

When, however, thin slips of this mass were dried on plates of glass, and rendered transparent by immersion in Canada balsam, a most intricate arrangement of capillary vessels was seen, ramifying and inosculating under various angles, and in a somewhat arborescent form, throughout the entire mass of the clot.

Thus, although the larger vessels might possibly have been the original vessels of transmission from the periosteum to the bone, yet it was evident that the whole clot was minutely organized with innumerable new and minute vessels; whose arrangement was so determinate and uniform, as to leave no doubt of the entire dissimilarity of the organization of this tissue from the periosteum on the one hand and the bone on the other." 213.

The coagulum must have been recent, and the fact, as well as the severity of the scurvy, are evidence enough of the debility of the patient.

Mr. Dalrymple thinks that the growth of malignant, and perhaps of the more simple, tumors affords another example of the tendency in weakened states of system to the organization of plastic effusions. It is well known that the former become developed much more rapidly towards the close of life, when the patient has been worn down by confinement, want of exercise, and long suffering. And it is not improbable that occasionally the latter or more harmless forms of tumor are converted into malignant during some sudden or gradual deterioration of the general health. But the tumors which result from the mere hypertropy of natural tissues, as the adipose, &c., must be excepted.

As it is from chronic inflammations that the main additions to the bulk of parts are made, Mr. Dalrymple attributes their persistence to cachexy. And he thinks that injection of new structures depends on want of tonicity of the capillaries of the old, and their consequent dilatation.

The Paper is an ingenious one, and the subject merits attention.

### III. A CASE OF RECOVERY FROM CUT THROAT, IN WHICH BOTH THE LARYNX AND PHARYNX WERE EXTENSIVELY OPENED. By R. A. STAFFORD, Esq., Surgeon to the St. Marylebone Infirmary.

J. S. aged 25, a servant out of place, with a razor, divided the larynx in full half its circumference, exactly between the os hyoides and thyroid cartilage, exposing its internal surface without wounding any important blood-vessel. He was brought into the St. Marylebone Infirmary by a policeman, October the 21st, 1839, and appeared in a very exhausted condition; having a feeble pulse and cold extremities. The wound had been dressed by another surgeon, and therefore it was not opened. Nutritious fluids were ordered, and, there being cerebral excitement, leeches to the temples, &c. were prescribed.

In the middle of the second night, whilst the back of the nurse who had been ordered to watch him was turned, he made an attempt to open the wound with an old blunt knife which he had secreted; and succeeded so far as to divide the sutures of the former wound, and to cut on into the pharynx. No vessel of any consequence was injured, and he coughed up only a little blood. The wound was brought together by two sutures, with the view of preventing the wide gaping which otherwise would have taken place, and it was lightly dressed. He breathed through the mouth.

From this period the food escaped through the wound in the pharynx, and it was necessary for him to be fed by an elastic tube being passed down the œsophagus into the stomach. The cerebral excitement and fever increased, and continued for several days. He was kept under strict restraint. Blood was frequently abstracted from the neighbourhood of the head: he was blistered and purged; took sudorifics; used freezing lotions and the cold douche; and at length symptoms of effusion into the ventricles and pressure on the brain, being by stupor and dilated pupils indicated, he was salivated by mercurial friction. The head-symptoms from this treatment gradually subsided, but he was left extremely weak. Quinine, wine and nutritious diet were administered, and his strength increased. The wounds now began to heal. The wound of the pharynx being the lesser, first closed up, and afterwards that of the larnyx. On the 9th of December the whole wound was completely cicatrized, but the voice of the patient was lost, and he could only speak in a whisper.

The man had twice before endeavoured to destroy himself, and, after quitting the Infirmary, he hung himself.

### IV. On the Structure of the Human Placenta, and its Connexion with the Uterus. By William Bloxam, Esq.

Mr. Bloxam has carefully examined the placenta. Mr. Bloxam gives a circumstantial account of his investigations, for which we must refer to the Transactions. His conclusions we transcribe. They are—

Firstly. That the blood enters the placenta through the short curling arteries of Hunter; and that although their size is insignificant compared with the venous system of the uterus, yet their relative number being greater, a full supply of blood is insured to the organ; whilst by the smallness of their calibre, they prevent the maternal circulation from expending its full momentum on the system of the child, under the accidental shocks physical and mental to which the mother is daily liable.

Secondly. That these vessels ramify on the spongy tissue of the placenta, and are there in apposition with the extremities of the umbilical veins.

Thirdly. That it is highly probable, that some of the properties of the maternal blood pass into the circulation of the fœtus by this means, and having fulfilled its functions in the fœtal œconomy, the residue is returned to the placenta by the umbilical artery.

Fourthly. That from the free terminations of the umbilical arteries it transudes into the interstitial structure of the placenta; which, it may be remembered, is continuous with the semilunar apertures on its uterine surface.

Fifthly. That these apertures are applied to the openings on the internal surface of the uterus, and furnish the channel by which the blood, or its principles, are restored to the system of the mother.

"As far as I am aware, there is no evidence of direct communication between the fœtus and its parent, furnished by the researches of comparative anatomists. Mercury, however, has been found in the vessels of the fœtus after having been injected from the uterus. Here, perhaps, the weight of the substance employed may have conduced to the result."

V. OBSERVATIONS ON INJURIES OF JOINTS, AND THEIR TREATMENT. By RUTHERFORD ALCOCK, Esq., K.C.T., &c. late Deputy Inspector General of Hospitals with the Auxiliary Forces of Portugal and Spain; and Lecturer on Surgery.

Mr. Alcock deserves the highest credit for communicating, as he does, the results of his experience in the recent revolutionary wars in Portugal and Spain. He has endeavoured to render the knowledge he has acquired available to the profession at large, and has published some highly interesting facts, as well as instructive observations.

The following memoir on Injuries of Joints deserves attentive perusal. We shall endeavour to select the more prominent points and lay them before our readers.

Mr. Alcock alludes in the beginning of the paper to excision of the articular ends of bones after injuries. And his remarks are valuable. He shews that this operation has not been sufficiently practised in the army.

*Excision of the Head of the Femur.*

"Excision of the head of the femur has been practised. M. Paillard, in his '*Relation Chirurgicale du Siège d'Anvers,*' gives an example of resection of the head of the thigh bone for a comminuted fracture of the neck,—six inches of the bone, including the head and neck, were removed with very trifling loss of blood. During the first few days, some chances of success were observed; but the limb quickly afterwards became gangrenous, and the patient died on the ninth day. Mr. White, of Manchester, was the first to propose this operation, about the middle of last century. One case only of its actual performance had been related before M. Seutin's, by an American, and was not considered, I believe, as very certainly authentic. The result of M. Seutin's case is not likely to lead many to perform an operation which, I cannot but think, very little calculated to save a useful limb under the most favourable circumstances. I believe, however, that Mr. White, Surgeon to the Westminster Hospital, has performed a similar operation for carious disease of the head of the femur, and with good result." 251.

*Excision of the Head of the Humerus.*

In wounds of the shoulder-joint, observes Mr. Alcock, Larrey speaks of ten cases in which he avoided amputation by extracting or removing the head of the humerus; one died of fever, two of scrofula, and one of plague. In some there was anchylosis, in others a species of joint.

In 1794, M. Percy showed, in Paris, nine successful cases, where the head of the humerus had been removed; and Larrey again relates a case where the top of the shoulder was struck by a four-pound shot, merely breaking the skin superficially, but fracturing underneath the head of the humerus, the scapular end of clavicle, the acromion and coracoid processes. He cut down upon the broken fragments, and removed them, including the head of the humerus. The wounds cicatrized, and the arm anchylosed at the shoulder, by the gradual approximation of the shaft. In another similar instance there was an artificial joint formed, and slight movement in every direction, although the arm seemed to have less strength than existed in the former case, where there was anchylosis. Mr. Guthrie also speaks of similar cases of cannon-shot injuries occurring in the British army; but all were fatal. He relates, however, several cases from musket-shot, where arms were saved by removing the head from the cavity, together with any other fragments of the neck.

These facts prove, as Mr. Alcock remarks, that excision of the head of the humerus will occasionally be successful even in injuries inflicted by cannon-shot, and may be applied with confidence to those resulting from musket-balls.

*Excision of the Knee-joint.*

Mr. A. reprobates this.

"To conclude at once with the articulations of the lower extremity, in reference to these operative means, I will observe that in injuries of the ankle, excision, whether of the astragalus or the end of the tibia, may form a resource; and examples are on record, when both the one and the other have been removed with success. If a shot, however, pass directly through the articulation, splintering articulating surfaces of both tibia and astragalus, I believe amputation to be almost invariably indicated. The excision or removal of the whole or portions of one of the articulating surfaces forming the ankle joint will more frequently be found expedient in the dislocations attended with laceration occurring in civil life, where it is often impossible to return the projecting parts to their proper position, than to gun-shot injuries more extensively shattering the bone, and contusing all the parts in the vicinity." 254.

*Excision of the Elbow-joint.*

Our author speaks favourably of this—a successful operation in civil practice.

*Excision of the Wrist.*

It is often, says Mr. Alcock, difficult to decide upon the course to be adopted in wounds and lacerations of this articulation. Patients occasionally recover from very severe injuries, such as the passage of a musket-ball through the carpal bones, or the laceration of a saw passing into the articulation with the hand. Yet these are cases at best eminently uncertain in their results; and although much may be adventured, but little should be promised or hoped. Excision of the end of the radius, the removal of a dislocated carpal bone, or of the head of a metacarpal bone, may occasionally be adopted, and with success, to prevent the loss of a hand. It is impossible, however, to predict, with any certainty, the degree of inflammatory and suppurative action which may ensue; and on this chiefly hangs the issue of the case.

If there be crumbling of one or more of the carpal bones, provided the tendons are not divided across, these fragments may be removed, and an attempt made to save the hand. But if there be comminution involving the greater part of the articulation, instead of its lateral extremities merely, it rarely happens that the tendons are not extensively torn across also ; and then amputation would better be performed at once, and no attempt made by excision to remedy the mischief.

The second part of Mr. Alcock's memoir is occupied with the general results of injuries of the articulations, with regard to their frequency in military practice, and to their treatment, progress, and termination. Mr. Alcock first supplies two tabular returns:

No. I.—Comprises the Results of Gun-shot Fractures, involving Articulations, Amputations,—including all in two Periods of about one Year each. Cases not operated on including the Series of Thirteen Months only.

No. II.—The Results of Gun-shot Fractures involving the Articulations, —in Cases Amputated,—in Cases treated without Operation. Including a Period of about thirteen Months.

Thus, says Mr. A., the Return No. 1, embraces all the cases occurring within a given period ; to which he has added all the amputations from such injuries, not only for the same but during several additional periods. As the periods embraced by the cases and the amputations do not therefore correspond, Return No. II, has been formed, giving all the cases treated or amputated within a given time, *and no others.*

Mr. A. has classed all injuries of joints under three heads. The first comprises those varieties of injury where in the majority of cases the limb may be saved, and where as a general rule it should be a principle of practice to attempt the cure.

The second contains what may be considered a doubtful or intermediate class. It comprises cases in which we find the difficulties both as to the diagnosis and line of treatment are considerably increased. They can be grouped neither like the first class, as injuries in which the attempt to save the limb may be made in the majority with good result: nor with the third class, where amputation is imperatively indicated from the first, and with as little delay as possible.

Mr. A. explains the principles of treatment which he has adopted, and which were applied to the cases included in the returns.

" In lacerated or incised wounds penetrating the capsule, it has been held matter of the highest importance to exclude the air, and secure, if possible, and by every means, union by the first intention. I have been led, by my observation of these injuries, to a conclusion not quite in accordance with this precept. Although it may safely be admitted as a general principle in surgery, that all wounds should be treated so as to procure the earliest possible union of their edges, this should be understood, in reference to wounds of joints, to apply only so far as the edges can be approximated without violence of any kind, constriction or pressure. I firmly believe that no pressure by bandages, compresses, or by means of strapping, can be applied in the first instance to injuries of joints, without doing mischief, and materially aggravating the inflammation, which, to some extent, must inevitably ensue.

I have found that cold, in the first instance, applied over the articulation, generally best assists in repressing and controlling the supervening inflammation, and if somewhat later it should become ungrateful to the patient's feelings, it may occasionally be exchanged, with advantage, for warm water dressings: or if the joint has assumed a puffy, swelled and unhealthy appearance,—a state often to be traced to the injudicious use of poultices,—a more tonic and stimulating mode of dressing will generally cause improvement. Of this kind of dressing, the best seem to me either a decoction of aromatic herbs, with the addition of a little wine, or warm camphorated or sweetened wine, which has not been freely adulterated with bad brandy, as are generally most of the wines consumed in England. Such dressing is not used in this country, but is frequently employed in the rest of Europe; and I have no hesitation in stating that I have seen the happiest effects from its use, when more emollient applications, such as poultices, certainly did not arrest, but, on the contrary, appeared to promote the '*engorgement*' of the limb. There is indeed a strong prejudice in this country against such applications, but founded upon theory rather than practice." 267.

Antiphlogistic treatment must be enforced, and leeches are more particularly necessary. Absolute immobility of the limb is to be secured. Yet suppuration too frequently occurs in spite of all treatment. The matter must be evacuated. Openings in dependent positions and free incisions, either in the vicinity, or if necessary, through the capsule, should be promptly and boldly practised, together with such regulated pressure above and below the articulation, as the state of the limb may indicate and allow, in order to counteract the tendency to spread and burrow.

The health of the patient must be, of course, attended to. If great prostration of strength, hectic, and diarrhœa set in, amputation may become necessary, whatever the original injury.

Perfect quietude during and for some time after the healing of the wounds, is indispensable. Then gentle friction and passive motion may be tried, very gradually. If the anchylosis resulting be partial, probably considerable motion may be recovered, and some force may be used; care being taken never to push these measures so far as to induce inflammation in structures still morbidly susceptible. If the anchylosis, on the contrary, be complete, no attempts of this nature should be made.

Mr. Alcock examines the tables of articular injuries in reference to the following circumstances.

1. Their proportionate numbers, in relation to other classes of injuries, and of the articulations with each other.

2. Mortality, absolutely and relatively. Number of amputations to which these injuries give rise, and proportionate numbers in different periods.

3. Causes of mortality, with regard to the whole number of deaths, and to the number of deaths from each articulation, considered in relation to amputations at the three periods—primary, intermediary, and secondary, and to cases treated without amputation.

4. Influence of external and collateral circumstances.

### 1. *Proportionate Numbers, &c,*

Table II. shews the proportionate number of articular injuries in about 1,800 wounded officers and men during little more than twelve consecutive months.

" The average may be stated, therefore, as between 4 and 5 per cent., or about $\frac{1}{22}$ of the whole number of 1,800 wounded; the injuries to the articulations counting 82. In a return already published of wounded *men* admitted into the General Military Hospital of San Telmo, in thirteen months, amounting to 1,350, the average is less. In the returns of the different actions it varies from 1 in 30, to 1 in 52; but in these are not included many of the worst joint cases, amputated on the field, which appear in the return as 'Field Amputations.' I consider, therefore, the Return No. II., formed with the greatest care and accuracy, to give the true average. In the returns already quoted of 1,351 wounded, at San Telmo, the proportion of injuries of the head was 7 per cent.; wounds of the trunk penetrating its cavities, between 4 and 5 per cent.; of fractures of the extremities, about 13 per cent., in which are included the joint cases; of severe wounds not in these classes, 33 per cent.; of slight wounds about 44 per cent.

With respect to the relative numbers in the different articulations, of the 82 recorded, nearly one-half are of the knee.

| | | | |
|---|---|---|---|
| Knee .. | 35: | proportion, 2·342: | mortality, 22 |
| Elbow .. | 19: | about .. .. $\frac{1}{4}$ .. .. .. | 5 |
| Shoulder | 11: | between .. $\frac{1}{7}$ and $\frac{1}{8}$ .. .. | 3 |
| Wrist .. | 7: | .. .. .. $\frac{1}{12}$ .. .. .. | 0 |
| Ankle .. | 6: | .. .. .. $\frac{1}{55}$ .. .. .. | 1 |
| Hip .. | 4: | .. .. .. $\frac{1}{20}$ .. .. .. | 3 |
| Total .. | 82 | Total .. | 34" |

2. *Mortality, absolute and relative.*

The mortality amounted to 34 cases in 82, including in the number treated those injuries which did not, in the first instance, implicate the structure of the joint, but only at some later period, by the progress of diseased actions spreading from the original site of injury. The number of cases in which the joint was primarily and directly implicated amounts to 65; 17 were only secondarily affected. Of the 65—

between $\frac{1}{5}$ and $\frac{1}{6}$, 12 were cured with more or less loss of motion and power, but all with some use of their limbs.

$\frac{1}{22}$, 3 intermediary* amputations were performed, and two patients died.

$\frac{1}{6}$, 11 secondary amputations; 5 deaths.

$\frac{1}{3}$, 21 primary amputations; 7 deaths.

Between $\frac{1}{3}$ and $\frac{1}{4}$, 18 died under treatment, while the attempt was being made to save the limb, either in the hope of success, or, more frequently, from the patient's refusal to submit to amputation.

Total 65

The result, therefore, stands thus—

33 recovered; 21 with loss of limb;
32 died; 18 without amputation;
7 after primary amputation;
2 after intermediary;
5 after secondary.

Total 32

* The term intermediary amputation refers to those performed between the third and twentieth days inclusive,—a period during which the febrile and inflammatory actions have commenced, rarely entirely subsided.

The mortality in the 3 classes stands thus, as regards the whole number of 65 :—

About $\frac{1}{9}$, or 7 died after primary amputation;

$\frac{1}{9}$, or 7 died after subsequent amputations;

Between $\frac{1}{3}$ and $\frac{1}{4}$, or 18 died during treatment without operation.

—

Total 32

—

These facts certainly tell in favour of primary amputation; for the 44 treated without it present a mortality of 25, more than one-half, whereas the primary amputations cause a loss only of one-third, although naturally performed for the very worst injuries: and while 12 only were cured without loss of limb, 18 died in the vain attempt to save, without, for the most part, offering any fair opportunity of remedying the evil by intermediary or secondary amputation.

Of the intermediary and secondary amputations, where treatment failing to save the limb, amputation offered the only ground of hope for life, 7 died out of 14, amounting to one half; but of the secondary amputations, properly so called, a fraction less than one-half were lost, 5 in 11. These cases form the forlorn hopes of surgery; all saved are snatched from nearly certain death.

Mr. Alcock says a few words on the comparative fatality of injuries of the different articulations.

*The hip* is more rarely the seat of direct injury from foreign bodies than any of the articulations. The result is generally fatal; three in four died; and in the fourth, where recovery took place, the joint itself, there is some reason to suspect, was but remotely affected.

*The shoulder* is rarely implicated directly by injury without a subsequent operation, amputation, or excision of the head of the humerus, being required. In 11, which occurred in the series of 82, only 2 were cured without amputation; 7 amputations were performed, 6 primary, and 1 intermediary; the latter was unfortunate in its result; all the primary recovered.

Amputation of the shoulder is a successful operation. In 9 cases there was but one death.

*Injuries of the knee* are the most numerous, and with the exception of the hip, the most fatal to life, and generally, at best, leading to the loss of limb: of 35 of the knee, 22 lost their lives, and of the remaining 13 who were saved, 8 lost their legs. The primary amputations amount to 9, and 4 died: they are decidedly more trying injuries to the system than those of the shoulder, not only by the double shock being greater, as proved by this result, but by the gravity of the actions, to which the original injury gives rise. Thus there were 3 intermediate amputations, and 9 secondary, and only 4 recovered out of the 12, or one-third, two-thirds dying.

*Injuries of the elbow* stand next in order of frequency, making a total of 19 cases, 5 of which were fatal; 10 were cured without loss of limb, but nearly all with anchylosis, complete or partial; 1 died during the attempt to save the limb, at the 120th day, of angina pectoris; 4 out of 7 primary amputations died, all with disease of chest or liver; 1 secondary amputation recovered.

*The ankle* is not often injured directly. There were but 6 cases; 1 died, and that from effusion into the serous cavities, anasarca, the limb erysipelatous and gangrenous; 3 required amputation, 1 primary, 2 intermediary,—all recovered.

*The wrist* is, upon the whole, more frequently injured, but in no proportion to the knee, and much less often than the elbow or shoulder. In 7 cases included in the series of 82, all recovered; 1 after secondary amputation.

Mr. Alcock has not included gun-shot fractures of the bones of the hands and feet in the returns of injuries of the joints—the articulating surfaces escaping serious damage surprisingly. And indeed the large bones may be splintered or perforated close to the great joints, without the latter being implicated.

### 3. *Causes of Death.*

Of forty-three fatal cases—

23 died under treatment for the original injury.
4 — after intermediary amputation.
5 — after secondary amputation.
11 — after primary amputation.
—
43 Total.

Of the 23 that died under treatment—

11 died of a wasting discharge and febrile action—hectic fever chiefly; the demands on the constitution great; the diseased action unconquered; disease from 8 to 72 days' duration;
2 with chronic tetanus, and one of these accompanied by hectic fever, the other by organic disease, namely, congestion of lungs, and abscesses of the left lobe of the liver;
1 from mortification;
1 delirium tremens;
1 with secondary hæmorrhage;
2 one with effusion into the serous cavities, and the other angina pectoris, both chest affections;
2 from the effects of other grave injuries;
1 from shock;
2 from causes not known.
—
23 Total.

Mr. Alcock does not conceive that injuries of the articulations are peculiarly prone to be followed by "purulent disease"* elsewhere. Nor do we see any reason why they should be so.

"In the injuries of the articulations for which amputation was performed, there were twenty deaths; and in seven the chief cause appeared, after death, to be purulent diseases in distant parts.

In gun-shot fractures not connected with the joints, the number of deaths after amputation was thirty-five. In eleven, the same effects were observed; one was of doubtful character, the cavity not having been examined; and two had

* By this expression we presume Mr. A. intends to designate the secondary inflammations and deposits.

pus in the blood-vessels, one in the femoral vein and artery, the other in the femoral vein alone.

Does this result depend upon the original injury? or is it to be considered as an effect of the amputation? The larger number after amputation must prove that the operation has a powerful influence, while the occurrence of precisely the same results in both sets of cases, in which no operation was performed, equally proves that amputation is, at least, not the only cause. Since both the injury and the amputation are each followed by these results, although in very different proportions, it seems most probable that in the greater numbers presented by amputations, the two shocks of the injury and the operation combine to produce this fatal effect.

This view is borne out by the fact, that such causes of death occur in the great majority after *primary* amputations, and not after those performed in subsequent periods, least of all in the secondary period.

In the injuries of the articulations, only one occurred after intermediate, and none after secondary amputation ; and in fractures only two cases, in like manner, after intermediate amputation. Thus the operations of the period nearest to the primary are the only ones that are followed by these peculiar effects in the series under consideration." 284.

Our author presents a resumé of the causes of death in the four classes, which shews that the chief danger and cause of death in cases treated to the end without operation, is hectic fever; and a variety of accidental or irregular complications, such as secondary hæmorrhage, epidemics, erysipelas, gangrene, &c., combined, form the remaining half.

The cases in which amputation is performed in the first instance, with fatal result, present a very different cause of mortality : the chief agent being purulent disease of lungs or liver, and occasionally inflammatory affections of the lungs or pleura. Fevers irritative or bilious destroy more than one-third.

The deaths after intermediary amputations are chiefly caused by febrile action, irritative or bilious; and in secondary amputations, the shock of the operations, hectic, and some accidental complications carry off the patients, already much reduced by suffering and the long continuance of wasting discharges. The results of secondary amputations, when fatal, and their causes of mortality, are in some degree assimilated to those predominant in cases treated to the end without operation.

Mr. Alcock furnishes three other returns, with the view of shewing the influence of favourable and unfavourable collateral circumstances. They exhibit in a striking manner the low mortality in the former, the high in the latter. But for this we must refer to the memoir itself.

The Third Part of that memoir is devoted to the,—

CLASSIFICATION OF KINDS OF INJURY IN WOUNDS OF THE ARTICULATIONS.

Mr. A.'s object is, if possible, to supply a *definition of those kinds of direct injury, in which we shall be justified in making the endeavour to save a moderately useful limb.*

I. After relating three cases, Mr. Alcock draws these conclusions from them.

1. A mere fissure of a joint, extending from a fracture, partial or complete, is not necessarily followed by severe, extensive, or destructive action in the structures of the articulation. If any other attendant circumstances, therefore, do not forbid, the attempt may be made to save the limb, with a fair prospect of a favourable result.

2. A foreign body, a musket-ball, for example, lodged either in the cancellated structure of the tibia or femur, may or may not penetrate the articulating surface, or project beyond it.

He continues.—" If it do not penetrate to the articular surface, it does not *necessarily* implicate the joint, or lead to any diseased action therein, and may at some later period be removed, if that be not possible at the time. If it do penetrate, but be smooth in surface, and not projecting beyond the level of the articulating surface, the same rule holds good, viz., that violent diseased action is by no means a necessary consequence, and I have been led to believe that in such a case, anchylosis, and a useful limb may, in many instances, be the result of careful treatment.

If the missile project, or if it be roughened, or cause any jagged projection of bone, the most destructive and rapid disease of the whole articulation follows, and in the knee especially, will inevitably lead either to amputation or death. In the elbow I have known such a case saved; in the knee never.

The following is the line of practice, therefore, which it seems to me should be acted upon. When any foreign body has penetrated the end of a bone forming an articulation, the surgeon should endeavour, by finger or probe, to obtain an accurate knowledge of its position. If he concludes, after such examination, that there is no projection into the articular surface, and it cannot be removed without great additional violence to the parts, such as burying the head of the trephine deep in the spongy end of a bone, the attempt should not be made, but the limb may be treated with a view to saving it. If we fail in this, a period will probably arise favourable to the performance of secondary amputation, and to its successful issue. If the ball has, on the contrary, penetrated into the joint, fracturing its way, and remaining either fixed or loose in the articulation, there is only one chance of safety for the patient's life, and none for the limb. Immediate amputation I believe to be the best and only judicious practice." 300.

II. Our author proceeds with the relation of two other cases, from which, and from some like them, he concludes that—

When the end of a bone, entering into an articulation is traversed by any foreign body or missile, more especially if it pass between the condyles of the femur or humerus, even though the integrity of the capsule, at one or more points, should be injured, if there be no detached fragment of bone, the joint, in many cases, may be saved; and the attempt may generally with propriety be made, when no other injury or unfavourable circumstance is superadded.

III. A successful case, in which a musket-ball apparently traversed the knee-joint, without occasioning fracture of the bones, succeeds. And Mr. Alcock remarks upon it:

" Upon cases of this class I have drawn the following conclusion. Wounds of the capsule, and even the traversing a joint by some missile or weapon, provided neither bone nor cartilage be seriously injured, do not require or justify amputation as a first remedy. The majority may be saved, even should the motion of the joint be more or less impaired; a useful limb may still be preserved.

Contrary to the general impression, I am strongly inclined to the conclusion, that injuries to joints are not fatal in proportion to the extent of surface laid open. The most dangerous of these wounds I believe to be punctured, or such wounds as a musket-ball creates,—a small, lacerated, and contused opening, with more or less mischief to the external parts. The most violent inflammatory action ensues in the highly susceptible synovial membrane, which, for a certain period, or until disorganization (the result of violent action) takes place, still retains its distinctive characters of serous or synovial membrane. Fluid is effused and pent up—the whole limb becomes involved—the system takes the alarm, and sympathises often to a certain extent. No kindly suppurative and granulating action takes place over the surface of the synovial membrane, altering its characters and susceptibility,—a result which follows not unfrequently in a wound laying a joint fairly open, quickly destroying, of course, the texture and character of synovial membrane, and leaving anchylosis as the only favourable result possible. But under such injuries, this is the happiest result we can ever look for; and the patient who so escapes has reason to be well satisfied that he has lost only the motion of a joint instead of a limb, or his life, or, as frequently must happen, the one first, and the other afterwards." 307.

IV. Mr. Alcock relates the case of a young gentleman, who met with a severe accident in leaping a railing, consisting of parallel bars. His foot caught, and his body was precipitated over, while he hung, with his leg engaged between the bars, for some seconds, before he could be disengaged. The knee was partially dislocated, but reduced at the time. Great extravasation of blood occurred in the ham, a tourniquet was applied, the femoral artery was tied in the upper part of the thigh, mortification followed, and the patient died on the tenth day. The crucial and posterior ligament of the knee were torn; the semi-membranosus muscle torn from its tendinous sheath; the popliteal artery and vein nearly torn asunder; the joint, and all surrounding parts, loaded with extravasated blood; the nerve uninjured.

Commenting on this case, Mr. Alcock is inclined to think that when there has been great violence offered to an articulation, sufficient to produce dislocation, and evident injury to a large blood-vessel in the vicinity, an incision should be made down to the vessels, and the nature and extent of the injury ascertained. If the artery alone be implicated, the capsule not extensively lacerated, nor blood extravasated within, a ligature may be placed above and below the ruptured point of the artery, and the case treated with a view to saving the limb. If any of these adverse circumstances be found, amputation should be proceeded with.

There will perhaps be a difference of opinion among surgeons on this point. An incision in the midst of a great mass of extravasated blood (and a moderate extravasation would scarcely justify it) would be far from unattended with serious danger of subsequent suppuration. And it would probably be likewise far from easy to discover or to tie the vessel under such circumstances. It must be owned that such a case is embarrassing.

We pass over a very interesting case under the care of that able surgeon Mr. White, of the Westminster Hospital, as well as a case in which the heads of two or three of the metacarpal bones were partially removed by a circular saw, with much laceration of the soft parts, and turn to the last case related by our author.

A gentleman, in 1835, was thrown out of his gig, and fractured the upper third of the ulna into the elbow joint. He was a stout muscular man, and considerable swelling supervening, before his surgeon saw him, the fracture did not seem to have been discovered until some degree of union had taken place, and that at such an angle, that a sharp peak projected at the posterior surface, rendering any attempt at flexion painful in the extreme, "cutting like a knife," as the patient described, from the stretching of the skin over the sharp end of bone. Gentle passive motion and friction had been employed when Mr. Alcock saw him. Mr. A. came to the opinion that mere ligamentous bands, uniting the two fragments at an angle, prevented the flexion of the arm, and that it required regulated, but considerable force, to elongate these. Before it could be attempted, the sharp projecting end of bone required removal. Sir A. Cooper concurred with Mr. Alcock, and the latter removed the projecting end of bone, and, as soon as the incision was soundly healed, he employed, morning and evening, a moderate degree of forcible extension, gaining by measurement, a very little each two or three days, but never proceeding so far as to excite inflammation. The case rapidly improved, and he has long recovered the perfect use of his arm, can carry his hand to the shoulder of the same side, row, &c., without pain or difficulty.

Mr. A. concludes, from cases of this description, that, wherever a partial anchylosis takes place, proving that there is not that kind of bony union, (of which there is a fine example among the specimens,) and no fragment of bone locks, so as to give the conviction, that unless it be broken, no farther progress can be made, the limited motion will generally be found to depend upon ligamentous bands or adhesions, which will elongate by the judicious use of force, to be employed twice daily, neither violent, nor long-continued, but so as perceptibly to gain by measurement, something, however little, each two or three days. In such cases, the gentle kind of passive motion, together with the frictions generally recommended, are perfectly inadequate, and altogether useless, except during the first few days after union, to facilitate the absorption of the effusion and thickening which may remain in the soft parts.

Mr. Alcock winds up this valuable memoir with a classification of injuries of joints, in reference to the principle of their treatment.

*First Class.*

1. Incised or lacerated wounds penetrating the capsule.
2. Penetrating wounds, with partial abrasion or contusion of articulating surfaces.
3. Simple fractures into joints, with more or less displacement, and subsequent confined ligamentous union.
4. Fissuring of articulating surfaces, from compound fractures, complete or partial in the vicinity, but without displacement of bone within the capsule.

"In this first class are included those cases, the great majority of which may be saved, and when it should be a principle of practice to attempt it. Of course, in the last division, fissuring from compound fractures, much judgment is required, to determine the curability of the fractured limb; the rule laid down is applicable only *quoad* the articulation. Moreover, in cases of fissuring from compound fractures, it will often happen, that only the head, or head and neck of the bone may be seriously damaged, and this, either with or without a foreign body lodging. Several fine specimens of this kind of injury are among my preparations. Here the limb may be frequently saved, as I have already shown, though not the joint, by excision of the head of the bone, or removal of the fragments." 318.

*Second Class.*

1. Foreign bodies lodged in the ends of bones, either not presenting in the articular surface, or on the same level and smooth.
2. Foreign bodies traversing the ends of bones, without detaching fragments from the articular surfaces.
3. Internal laceration of ligamentous structure, with lesion of blood-vessels, and with or without temporary displacement of articulating surfaces.
4. Separation of shaft from epiphyses with possible laceration of capsule, but not extensive.

This is a sort of *intermediate* class between those in which the rule is to attempt to save, and those in which the rule is the reverse.

*Third Class.*

1. Compound fractures into joints, with displacement and roughened edges.
2. Foreign bodies projecting into articulation, or traversing with extensive injury to structure.
3. Lacerated wounds of capsule, with much contusion and injury to internal structure of articulation, and with extravasation of blood into the joint.

The plan, here, should be to amputate without delay, unless excision of the head of the bone be deemed advisable. In the second kind of injury in this class Mr. Alcock has never known recovery to take place, when any of the large articulations were affected, rarely even when the injury was in the smaller.

"An extensive injury to a joint will sometimes destroy the patient by the shock. Or a wound of an articulation may be complicated with some other grave injury; such as a penetrating wound of the chest. Unless the second wound be of fatal character, I do not think it should prevent the necessary steps being taken with reference to the joint. In one such case, I amputated at the shoulder joint, and although the patient ultimately died, having been seized with a bilio-remittent fever, which attacked nearly all the amputations of the period, he did not die till the thirty-first day: the *lung, however, presented no trace of active disease,* although the wound was in the chest, the morbid actions seemed to have been expended upon the vicinity of the articulation.

The most excessive action sometimes follows a slight injury, and I have known erosion of the cartilages take place in five days. In another case, a superficial wound of the inside of the knee seemed to develope the most frightful actions, local and general, destroying the limb with suppurative disease, and consuming all vital power by fever. The ball which had curved down towards the ham-strings, but still superficially, I removed on the sixth or seventh day, and he died about the fifteenth.

At other times there will be, comparatively, little action in the joint itself, and the whole mischief be expended below; or again, I have known a joint filled with pus, but no erosion or alteration whatever in the cartilages.

It is worthy of remark, also, that a joint often becomes secondarily affected, and with so little attendant pain, as to escape observation for some time." 322.

We think Mr. Alcock entitled to the thanks of the profession for this very able and useful paper. It will tend to direct attention to injuries of the joints and to give precision to our views in treating them.

### VI. On Aneurysms, and especially Spontaneous Varicose Aneurysms of the Ascending Aorta, and Sinuses of Valsalva. With Cases. By John Thurnam, Esq.

Mr. Thurnam, a gentleman well known to our readers by his zealous investigation of several pathological points, has contributed an able memoir on aneurysm of the ascending aorta. We shall notice the principal points in it. Mr. Thurnam observes :—

"In consequence of witnessing a remarkable case of aneurism of the right aortic sinus of Valsalva, which opened into the right ventricle, (see case 7,) my attention became directed to the probable effects of aneurisms of each of the three aortic sinuses, especially in relation to the particular cavity, whether of the heart, of a blood vessel, or of the pericardium, with which, in case of their becoming ruptured, they would probably communicate. I have endeavoured to determine this question, by two modes of investigation : 1stly, by anatomical examination and experiment, and 2ndly, by comparing the results of cases of aneurism thus limited, whether recorded in the annals of medicine, preserved in museums, that I have visited, or occurring under my own observation." 325.

Omitting a brief, but sufficient, account of the relations of the ascending aorta, we pause at a description of the sinuses of Valsalva—three roundish dilatations corresponding to the three semilunar valves.

"These sinuses have, hitherto, not been generally indicated by names ; for the sake, however, of convenience in this paper, I shall speak of them as the right, left, and posterior aortic sinuses. By the right and left, I mean the two sinuses which are seated anteriorly, and from which, respectively, the right and left coronary arteries arise ; and by the posterior, I intend that which does not give origin to any coronary vessel. The term posterior, which is sanctioned by the authority of Valsalva himself, appears to me to indicate the position of the sinus last alluded to, better than that of 'intercoronary,' which has been applied to it by M. Bizot. All the sinuses are imbedded in the soft fat, generally found at the base of the heart ; but the right is seated more superficially than either of the other two.

By passing needles through the coats of the aorta, in the situation of the sinuses, I have ascertained, that when affected by aneurismal or other disease, any one of them might become ruptured into the pericardium ; but that the right is more liable to do so than either of the others. By the same means, I have found, firstly, that the right sinus might form a communication with the top of the right ventricle, with the pulmonary artery, or with the right auricle at the mouth of the appendage ; secondly, that the left aortic sinus might become ruptured into the left auricle, the left ventricle, or the pulmonary artery ; and thirdly, that the posterior aortic sinus might open into either the right or left, though more readily into the right auricle. I have likewise found that the ascending aorta, for some distance above the valves, has very similar relations to the adjacent parts as the sinuses themselves ; but that, as indeed is obvious from simple inspection, the relations of aneurisms of the higher part of the ascending aorta, are with the superior vena cava on the right ; the pulmonary artery on the left ; the pericardial cavity in front ; and with the right pulmonary artery and veins, and with the right, and in a less degree, the left bronchus behind." 327.

### *Liability of the respective Sinuses to Aneurysm, and their relation to adjacent Parts.*

The Paper contains references to eighteen cases of aneurysm, more or less accurately limited to the aortic sinuses. In two of these cases there were aneurysms so limited in two of the sinuses; and, in another instance, the three sinuses were all affected. Of these twenty-two aneurysms, twelve were seated in the right, four in the left, and six in the posterior aortic sinus. Thus the right sinus *seems* more liable to aneurysm than either of the others. Of the twelve aneurysms seated in the right sinus, two were of an incipient form, and, doubtless, projected into the pericardium; two had ruptured into that cavity; six projected into, and one had actually formed a communication with the highest part of the right ventricle. Of the four aneurysms seated in the left sinus, one had become adherent to the left auricle; and another had formed a large tumor in the upper part of the left ventricle. Of the six aneurysms seated in the posterior sinus, two were incipient, and probably projected into the pericardium; one projected, as a round tumor into both the auricles, but principally into the right; and one probably had become ruptured into the sinus of the right, and another into that of the left auricle.

Mr. Thurnam thinks that that portion of the ascending aorta which is immediately above the valves, is probably even more liable to circumscribed true aneurysm than are the sinuses of Valsalva themselves. But he is inclined to believe that those portions of the ascending aorta which are directly above the attachment of the semilunar valves, and which consequently are seated above and between the aortic sinuses, are more liable to the formation of aneurysm than are the intermediate portions. He is able to refer to ten cases thus seated. Of the eight aneurysms seated above the attachment of the right and left aortic valves, three had become ruptured into the pericardium; one projected, and threatened to burst into the right, and another into both the right and left ventricles of the heart: whilst, in four cases, the aneurysmal sac was ruptured into the pulmonary artery. In one case seated above the insertion of the right and posterior valves, the sac had become ruptured into the right auricle; and in another, above that of the posterior and left, the sac appears to have projected into the sinus of the left auricle, and had become ruptured into the pericardium.

*Varicose Aneurysm.*—This, and aneurysmal varyx have till lately been regarded as necessarily preceded by a wound of the artery and vein. But says Mr. Thurnam—

"Previously, however, to the publication of the memoir of M. Breschet, Mr. Syme had narrated an interesting case of spontaneous varicose aneurism in the abdominal aorta and cava. Two important cases likewise, the one by Mr. Porter, and the other by Mr. J. G. Perry, in which spontaneous varicose aneurisms existed in the popliteal and femoral arteries and their accompanying veins, have also been published; for the precise nature of the latter case having been established by dissection, that of the former appears to me no longer conjectural." 330.

Mr. Thurnam points out the peculiar liability of the ascending aorta to varicose aneurysm, from its anatomical relations, and he adds,—

"It may perhaps be necessary in this place to meet the objection that very possibly will be made, that to call an aneurismal sac which has ruptured into one of the right cavities of the heart or into the pulmonary artery, a spontaneous varicose aneurysm, is a 'pathological transcendentalism,' 'founded on a farfetched, though ingenious, analogy.' I have, therefore, been gratified to find since a great part of this paper was written, that Mr. Smith of the Richmond Hospital, Dublin, has taken a similar view of the subject; and has anticipated the probable occurrence of such a lesion as spontaneous varicose aneurism, in the very centre of the circulating system. In remarking on a case to which I shall have to allude, and the preparation of which I have had the advantage of inspecting in his company, Mr. Smith observes, 'had the sac yielded where it projected into the right ventricle, there would have been formed a varicose aneurism of a new and extraordinary description, and I should think not of necessity at once fatal.'

But it is not merely on speculative grounds that I would contend for this view of the subject; for, as I hope to show, there is somewhat more than a general analogy between the phenomena presented by the ordinary varicose aneurysm of the extremities and those observed in the cases under consideration." 332.

Mr. Thurnam details in succession the following cases—

1. Spontaneous varicose Aneurysm of the Abdominal Aorta and Vena Cava.
2. A similar case.
3. Spontaneous varicose Aneurysm of the abdominal Aorta and Vena Cava. Death in a few hours after the probable period of formation of the opening.
4. Spontaneous varicose Aneurysm of the ascending Aorta and Superior Vena Cava.
5. Spontaneous varicose Aneurysm of the ascending Aorta and appendage of the right Auricle.
6. Spontaneous Varicose Aneurysm of the three Sinuses of Valsalva, right Auricle and Termination of the Superior Cava.
7. Spontaneous Varicose Aneurysm of the Right Aortic Sinus and summit of the right Ventricle of the Heart.
8. Spontaneous varicose Aneurysm of the ascending Aorta and Pulmonary Artery. Death nine hours after the probable period of formation of the opening.
9. Spontaneous varicose Aneurysm of the ascending Aorta and Pulmonary Artery.
10. Spontaneous varicose Aneurysm of the Aorta and Pulmonary Artery. Death in about twelve hours after the probable period of formation of the opening.
11. Spontaneous varicose Aneurysm of the Arch of the Aorta and the left Pulmonary artery, corresponding to the situation of the Arterial Duct.
12. Spontaneous varicose Aneurysm of the ascending Aorta and Pulmonary Artery. Sudden death at the time of the probable formation of the opening.

Such of our readers as are anxious to peruse these cases we must refer to the volume of Transactions that contains them. We proceed to the conclusions which Mr. Thurnam draws from them. We should observe, that, in addition to the twelve cases whose titles we have given, Mr. T. describes, in a note, the preparations from six others, in which spontaneous varicose aneurysms had existed. Of all these, three were seated in the descending aorta and vena cava. The others, excepting one in the arch, were all seated in the ascending aorta or its sinuses; and communicated, one with the superior vena cava, two with the right auricle, one with the right ventricle, and eleven with the pulmonary artery.

*History.*—The twelve patients were all of the male sex. Two were from twenty to thirty, four from thirty to forty, two from forty to fifty, three from fifty to sixty, and one sixty years of age. One was by profession a merchant, one a coachman, much exposed to wet and cold, one a butler, one a baker, one a tinsmith, and another a porter: the three latter were all accustomed to lift heavy weights. The profession of the six other patients is not stated; excepting that one of them was a gentleman.

The habits of the merchant and baker were temperate; of the coachman, tinsmith, and another, decidedly intemperate; whilst those of the other seven are not stated. One had sustained an injury on the back two years and a half before; another had complained, for two years, of severe pain stretching across the loins; and two had suffered from acute rheumatism, the one ten, and the other thirteen years before the appearance of symptoms of aneurysm. One had had an attack of hemiplegia nine years previously, and had complained of pain and swellings in the hands and feet, for two years before the attack; one had suffered from dyspnœa and palpitation, during the greater part of his life; one from slight bronchitic symptoms and præcordial uneasiness for some months; and another from marked symptoms of disease of the heart, during six months.

Mr. Thurnam properly observes that the foregoing features rather bear on the etiology of aneurysm generally, than on that of spontaneous varicose aneurysm in particular.

Mr. Thurnam proceeds to observe that the communications between the artery and vein would seem to be formed in one of two principal modes. In six of the cases, the opening into the venous system seems to have been formed very suddenly; in consequence, in four of them, of some more or less unusual exertion or effort, previously to which the patient had been in ordinary health. In two of these cases, the effort consisted in raising heavy weights; in one, in playing with children, after a fatiguing walk, which had been followed by a hearty meal; and probably in the other, it was the result of the action of drastic purgatives.

In these cases the aneurysmal sacs were, no doubt, ruptured. In the first of the four, the symptoms consisted of a sensation of something giving way in the chest; in the next, of faintness, dyspnœa, and palpitation, with pain, and a sense of something cracking about the heart; in the next, of vomiting, oppression in the chest, and a constant desire to cough, with bloody expectoration; and in the last, of a sudden increase of dyspnœa and feebleness of pulse. In two cases likewise, where the patient does not seem to have been exposed to any particular exertion, the preternatural communication was evidently formed in an equally sudden manner; and was indicated, in the one, by great depression and violent vomiting; and in the other, by severe dyspnœa and insensibility.

In most of the cases, however, the openings probably resulted from a gradual softening, or ulceration of the walls of the sac. For, in three, the mode of attack is not particularly specified; in another, however, we find that there was no sudden seizure, but that the patient suffered for some time, from pains in the neck and shoulders, which ceased more or less completely, as the symptoms of the varicose communication appeared. In the three cases in which the aneurysms were seated in the descending aorta, the symptoms were preceded by pains in the region of the back; and, in the two former of these also, there appears to have been no sudden seizure.

*Symptoms.*—Mr. Thurnam regards these in connexion; 1stly, with the external surface and system generally; 2ndly, with the respiration; and, 3rdly, with the state of the heart and great vessels.

1. Excepting, writes Mr. Thurnam, two or three cases in which the patients died almost immediately, the external surface in all presented very decided signs of an obstructed circulation. In four, the animal heat appeared to be more or less remarkably deficient. In six the surface, especially that of the face, was livid or bloated; and in one of these the livor extended to the mucous membrane of the fauces and soft palate, the colour in the face being of a peculiarly pallid character. In one of the cases, in which the descending aorta and inferior cava were the seat of the lesion, the veins of the abdominal parietes were large, tortuous and distended. In the two cases in which the opening was in the superior cava, many of the superficial veins of the upper half of the body, particularly those of the face, neck, front of the chest, and back, were permanently distended, and almost varicose. A similar but more general condition of the superficial and other veins, also existed in two other cases.

Dropsical infiltration of the surface was the most uniform symptom noticed, and was present in all the cases, eleven in number, in which the symptoms are detailed, excepting three, in which death very speedily occurred after the formation of the varicose opening. The anasarca in all was very decided, and advanced very rapidly: in one case, it made its appearance on the twentieth day from the date of the intervascular communication. It involved all those parts of the body, the veins of which were distal to the opening in the venous system. Thus in the case in which the aneurysm opened into the superior cava, and in that in which it opened into the top of the right auricle, the anasarca of the face and arms was remarkably contrasted with the uninfiltrated state of the lower half of the body. In two cases, in which the abdominal aorta and inferior cava were the seat of the varicose aneurysm, the anasarca of the legs and lower half of the body was equally strongly contrasted with the free, and in one case, emaciated state of the arms.

In three other cases, in which the aneurysm opened into the appendix of the right auricle, the right ventricle, and the pulmonary artery respectively, the venous system of the whole body being distal to the varicose orifice, we find that the dropsical infiltration was little short of being universal; though, as in all cases of general dropsy, the lower extremities were most decidedly affected. There was, likewise, more or less ascites in these three cases. The extreme debility, also, with emaciation, which was noticed in two of the cases, ought, probably, to be regarded as a symptom proper to the lesion under consideration. In one instance, sloughy sores formed on the shins; and in another, sloughing of the scrotum supervened on the operation of acupuncture.

2. Excepting two cases in which this symptom is not mentioned, there was more or less dyspnœa in all; but in four the oppression and difficulty of breathing were not very severe, excepting, in most of them, towards the fatal termination, and were chiefly noticed after exertion. In the other six cases the dyspnœa was extreme, and amounted to orthopnœa, in the two former, of a most aggravated character, the patient dying from a slowly developed apnœa (asphyxia). There appears to be reason for believing that the urgency of the symptoms generally, and especially of the dyspnœa, is in proportion to the size of the opening into the vein, and to its proximity to the lungs; and, consequently, that this symptom, *cæteris paribus*, is more severe when the aneurysm communicates with the pulmonary artery, than when it opens into the inferior or superior cava. Cough is stated to have been present in every case but five; and in several instances it was particularly distressing. It was almost uniformly attended by expectoration; and in two cases only, where there was cough, is this symptom not mentioned. The sputa were more or less mixed with blood in three of the cases.

But to appreciate the cause of the dyspnœa, it is necessary to look to the condition of the lungs and pleuræ. In one case, the pleuræ were uniformly adherent, and in seven hydrothorax, to a greater or less extent, was present; though, perhaps, in four cases only, was the fluid in such quantity as to have materially affected the respiration. It is most probable, that this effusion into the pleural cavities is to be regarded as a consequence of the varicose communication. In one case, there was pulmonary apoplexy; but, excepting for the most part, slight congestion and œdema of the tissue of the lungs, in five or six instances, these organs appear to have presented no other lesion. It may then be observed, that the severity of the dyspnœa, &c., in very few of the cases, bore any proportion to the degree of pulmonary complication; consequently we must, in the main, attribute these symptoms to the varicose aneurysm.

3. In five of the cases, continues Mr. Thurnam, there were palpitations of the heart, and, in one, præcordial pain. As, however, these were precisely the cases in which the heart itself presented the most decided traces of disease, in the shape of hydro-pericardium, dilatation and valvular lesion, it is doubtful how far we can look upon the symptoms alluded to, as directly belonging to the varicose communication. The pulse presented distinctive characters in all the cases, eight in number, in which it is noticed; if we except one, in which it is briefly stated to have been "hard," a character which it is difficult to reconcile with a communication between the ascending aorta and vena cava. In four of the cases, the pulse was decidedly "jerking;"—the terms, "vibratory," "hæmorrhagic," "resilient," and "thrilling," applied by three of the observers, evidently referring to one and the same character. In another case, it was stated that there was a distinct interval between the impulse of the heart, and the pulse, as felt at the wrist. In the other two cases, the pulse was chiefly distinguished by being extremely feeble; and this was likewise the case in two of the instances in which it was jerking. In one, the pulse was much weaker in the left than in the right wrist; and in three cases, it became either intermittent or irregular in the progress of the disease.

*Physical Signs.*—" In the first only of the two cases of varicose aneurysm of the descending aorta and inferior cava, were the physical signs noted; and in this they consisted of a pulsating tumour of the abdomen, with an incessant whizzing sound proceeding from the same part, and audible both to the patient himself, and to those around. In the case in which the aneurism opened into the superior cava, there was a distinct impulse detected under the right clavicle, and on the right border of the first piece of the sternum; and a loud murmur was also heard in the same situation. In the case in which the aneurysm communicated with the appendage of the right auricle, a distinct pulsation and a bellows murmur were also perceived on the right of the sternum.

In the instance in which the varicose communication existed between the ascending aorta and the upper part of the right auricle, there was a loud double bellows' sound, which was particularly heard over the upper part of the sternum; the systolic portion of the sound was more prolonged, the diastolic sharper and shorter.

The physical signs, as observed by myself, in the case in which the aneurysm opened into the summit of the right ventricle, differed remarkably from the preceding, as to the situation in which they were heard. There was dullness on percussion in the præcordial region, which extended to the level of the second rib. The healthy sounds of the heart were scarcely audible, and that only in the arteries of the neck. Throughout the præcordial region, and indeed over nearly the whole thorax, a continuous sawing sound was heard. This sound was loudest during the systole, less loud during the diastole, and still less so during the interval: it was most distinct in the second intercostal space, about an inch and a half from the sternum; where, in a spot that might be covered by a shilling, it was intensely loud and superficial; and in the same spot there was a most distinct and superficial purring tremor. In this case it is to be recollected that the heart was displaced somewhat to the left, by dropsy of the right pleura. Dissection proved that the spot where the murmur was heard and the tremor felt most distinctly, corresponded precisely to the situation of the varicose orifices.

The physical signs in the first of the cases of varicose aneurysm, connected with the pulmonary artery, were not at all noticed, but it is to be observed that this occurred prior to the great discovery of Laennec. In the next case, in addition to increased though not forcible impulse, and dullness on percussion, in the region of the heart, there was a loud blowing sound heard over the front and back of the chest, but most distinctly at the middle of the sternum. In this case also the murmur would appear to have had a continuous character. In the remaining cases the physical signs do not appear to have been noticed, at least not after the formation of the varicose opening." 369.

There were in several of the cases other symptoms not of so specific, or distinctive a character—such as dizziness, a blood-shot state of the eyes, impairment of speech, hæmaturia, and melæna.

*Pathology.*—When, says our author, a communication exists between the ascending aorta and an adjoining part of the venous system, the arterial blood, in consequence of the greater power of the left ventricle, is propelled through the opening, becomes mixed with the venous blood, and is carried forward with it to the lungs. The pathological effects consequently resulting, are hence obviously referrible to one of three circumstances.

In the first place; a portion of arterial blood is abstracted and regurgitated from the arterial system; and the arteries, consequently, are imperfectly filled. As a consequenee, the pulse is feeble, and peculiarly jerking; the surface, and especially the countenance, loses the ruddy hue of health; the animal heat is diminished; and, the various organs being imperfectly nourished and stimulated, there arise emaciation, debility, loss of muscular power, with a disposition to syncope, to gangrene, and even to softening of the heart and internal viscera.

Secondly; the stream of arterial blood, which is constantly passing into the venous system, acts as a direct and powerful impediment to the return of the venous blood from the veins distal to the varicose orifice; and this is an effect which, in some cases, is assisted by the pressure of the aneurysmal tumor. Hence arise, in the parts so situated, livor of the skin and mucous membranes; venous congestion of the glandular system, especially the liver; engorgement and dilatation of the right cavities of the heart; distention, and a varicose condition of the sub-cutaneous and deep-seated veins; passive hæmorrhages; dropsical effusions, especially in the shape of anasarca; and venous congestion of the brain, with comatose and apoplectic symptoms.

Thirdly; the circulation through the lungs of a portion of already arterialized, in a state of mixture with the impure venous, blood, and in vessels not intended for its reception, acts, in all probability, as an abnormal stimulus or irritant to the pulmonary organs. We consequently have dyspnœa, cough, and the secretion, from the air cells and bronchial tubes, of a more or less viscid mucus, often tinged, or even mixed, with blood; and the lungs, after death, are frequently more or less congested, and may even be the seat of apoplectic effusions.

Mr. Thurnam says a few words on the rationale of the physical signs of varicose aneurysm of the aorta. What he does say appears to us exceedingly judicious.

"As a consequence of the superior force of the left venticle, the arterial blood is doubtless propelled through the varicose orifice, and so produces the murmur. During the systole of the heart, the current through the orifice is the strongest, and the sound consequently is then the loudest. During the diastole, in consequence of the elastic reaction of the arterial system on its contained blood, a less powerful current is propelled through the opening, and at that time, a somewhat weaker murmur is heard. This reaction of the arteries, however, is in operation, not only during the diastole, but also during the interval, and, in fact, until it is overcome by the succeeding ventricular systole; consequently, though the current is stronger at the commencement of this reaction, and synchronously with the diastole, yet is it also continued during the interval. Hence, the murmur is a continuous one; it being present, though much weaker, during the interval between the diastole and the succeeding systole. The same circumstances which produce the murmur, of course occasion the purring tremor. I think there can be no doubt but that the extremely loud and distinct character of the murmur and tremor, are due to the generally small varicose apertures, through which the blood is propelled into the vein or right cavity of the heart; and that their intensity will be found to be in a direct ratio to the smallness of the aperture, and to the proximity of this to the walls of the chest. Like all other organic murmurs, the sound will be heard the loudest over the orifice in which it occurs; and, like them, will be propagated in the direction of the circulation beyond. Consequently, when the aneurysm opens into the vena cava superior, or the right auricle, it is on the right border of the upper half of the sternum, that the sound will be chiefly heard, and the tremor felt; but, when the communication is with the summit of the right ventricle or pulmonary artery, it is on the left border of the upper third or half of the sternum, that the sound and tremor will be the loudest and most distinct." 373.

*Diognosis.*—Mr. Thurnam thus enumerates the diagnostic signs:—

*General signs.*—1. Severe and rapidly advancing anasarca, of such portions of the body as are below, or the venous system of which is distal to, the varicose orifice. When the varicose aneurysm is between the descending aorta and inferior cava, the legs, scrotum, and lower half of the body; when between the ascending aorta and the superior cava, the arms, face, and upper half of the body; and when between the ascending aorta and one of the right or left cavities of the heart, or the pulmonary artery, the whole of the body is the seat of the dropsical effusion.

2. Livor of the face particularly, but likewise, in a less degree, of all such portions of the body as are below the varicose orifice.

3. A distended, and even varicose, condition of the subcutaneous and other veins, distal to the orifice.

4. Dyspnœa; often amounting to orthopnœa and terminating in apnœa.

5. Cough, with expectoration; especially if the sputa be bloody.

6. A remarkably jerking, and in some cases, very feeble pulse.

7. Emaciation, debility, loss of muscular power, deficient animal heat and sensorial disturbance, may be looked upon as somewhat less frequent and certain signs.

*Physical signs.*—8. A superficial, harsh, and peculiarly intense sawing or blowing sound, accompanied by an equally marked purring tremor, heard over the varicose orifice, and in the current of the circulation beyond it; this sound is continuous, but is loudest during the systole, less loud during the diastole, and still less so during the interval. The characters of the sound, as regards intensity and continuousness, will probably altogether distinguish it from any that is heard in ordinary cases of aneurysm, or valvular disease of the heart. In the case of a varicose communication between the aorta and superior cava or right auricle, when there is no displacement of the heart, the sound will be heard and the tremor felt, along the right border of the sternum; and will, generally, be the loudest about the second right intercostal space. When, however, the aneurysm opens into the pulmonary artery or summit of the right ventricle, the corresponding points on the left side will be the seat of the murmur; though this may, probably, sometimes be heard more distinctly nearer to, though still to the left of, the centre of the sternum.

When the history shows that the foregoing signs have been developed soon after some unusual effort, especially if that were attended by pain in the præcordial region and a disposition to syncope, the evidence of a varicose aneurysm of the ascending aorta is rendered nearly indisputable.

*Prognosis.*—This may easily be stated—death. The duration of the disease, dated from the formation of the varicose opening, appears only to be indicated with precision in four of the cases. In four of them, the cases had a general aspect, very much resembling that of rupture of the heart; and the patients survived, in one case only four minutes, and in the other three from nine to twelve hours each. In one of the other cases the patient lived a month; and in the other, eleven weeks and two days. The probable duration of the disease in the five remaining cases, was in one, about two months; in two, about four; in one, five; and in another, ten months.

*Treatment.*—Not much need be said on this. It must be palliative, and can consist only in local bleeding for the relief of congestions, diuretics, and slight diffusible stimuli.

### *Analogy with the ordinary Forms of Varicose Aneurysm, &c.*

"According to M. Breschet, the latest and most accurate writer, at length, on this subject, the rational signs of varicose aneurysm of the extremities, consist of numbness, loss of power, diminished heat, a blueish or slightly violet tinge of the skin, and a small and feeble pulse (which Scarpa states to be likewise vibrating), in that portion of the limb which is beneath the aneurysmal tumour. Now the only signs of spontaneous varicose aneurysm of the aorta, that we should at all look for in cases seated in the extremities, and which are not mentioned, are the distended and varicose state of the veins, the œdema, and the symptoms referrible to the respiration. But when we recollect the free inosculations which exist between all the principal veins of the extremities, and the absence of such in the vena cava, right cavities of the heart and pulmonary artery, it is easy to perceive why œdema, and a distended or varicose state of the veins below the opening, should be absent in varicose aneurysms of the extremities; and why they should be present in the same lesion, when situate in the ascending or descending aorta. Again, the comparatively very small quantity of arterial blood which, in the former cases, circulates through the lungs, and the greater distance from these organs at which it enters the venous system, afford a sufficient explanation of the absence of dyspnœa, cough, and the other symptoms of pulmonary disturbance.

With respect to the physical signs of varicose aneurysm of the extremities, it may be observed that by all authors who have treated of them, from Dr. William Hunter downwards, they are stated to consist of pulsation and purring tremor, in the situation of the tumour, accompanied by a decided, and, in most cases, *very loud* murmur, which is said, by some, to be propagated up the vein. The murmur, which has been variously described as a humming, whizzing, hissing, or roaring sound, is in some cases not merely audible when the ear is applied over the tumour, either with or without the stethoscope; but is also, as in that of Mr. Syme, and in the fifth reported by M. Breschet, audible to the patient himself, and even to those at some distance around him. In many of the cases also the sound is described as being alternately louder and more feeble, synchronously with the motions of the heart, so that a *continuous* sound would appear to have been present." 379.

### *On Aneurysms of the ascending Aorta, ruptured into the left cavities of the Heart.*

It has been seen, from anatomical examination and experiment, that aneurysmal sacs, when situated in certain of the sinuses of Valsalva, or in certain portions of the ascending aorta, would be likely to form communications, not with the right, but with the left cavities of the heart. In such cases, the lesion cannot, of course, be denominated a varicose aneurysm, though both the general and the physical signs would, probably, have much analogy to those belonging to such cases. Mr. T. relates two cases—the first, one of aneurysm of the posterior aortic sinus, communicating with the left auricle—the second, one of aneurysm of the left aortic sinus, projecting and threatening to become ruptured, into the left ventricle.

Mr. Thurnam concludes his highly interesting and valuable paper by a note:—

"Whilst this paper was passing through the press, two additional cases of spontaneous varicose aneurysm were published. The one was seated in the ascending aorta and pulmonary artery, and the other in the common iliac artery and vein: they were communicated, the former by Mr. Smith, the latter by Mr. Adams, to the Pathological Society of Dublin, in April last." 384.

So that, from the publication of eight cases, in as many months, this lesion must be looked on as more than a pathological curiosity.

### VII. CASE OF A RARE SPECIES OF HYDATID, (THE ECHINOCOCCUS HOMINIS,) FOUND IN THE HUMAN LIVER. By T. B. CURLING, Esq.

A muscular man, aged 71, died March 18, 1841, in the London Hospital. There were disease in the left sterno-clavicular joint, congestion of the lungs, granular kidneys, and stricture of the urethra.

"On opening the abdomen, my attention was attracted by a cyst connected with the margin of the left lobe of the liver. It caused a tumor projecting from the gland, which was slightly adherent to the peritoneum, covering the pylorus and commencement of the duodenum. This cyst was of an oval figure, and measured about 2¾ inches in its long diameter and 1¾ inches in the other. A section displayed an old hydatid cyst varying in thickness in different parts, and fibro-cartilaginous in structure, lined by a soft loose albuminous membrane enclosing a large number of separate hydatid cysts of various sizes from that of a pea to that of a large cherry, surrounded by and floating in a transparent fluid. These cysts which were exactly similar in structure to the *acephalocyst*, being white, opaque, and divisible into layers, were also found to contain a perfectly limpid fluid which remained unaltered in appearance after one of the hydatids had been immersed for several minutes in boiling water. On opening a cyst there escaped a large number of small white particles, some of which were found floating in the fluid within; whilst others were in contact with the inner surface of the membrane composing it. The latter appeared like grains of white sand thickly studded over the interior of the cyst. On examination in the microscope, these little bodies were ascertained to be the vermiculi of the *Echinococcus*, all the characters of which were very distinctly perceived. They presented various appearances, according to the position of the animal submitted to examination. In some, of which we had a lateral view, we could see the prominent head surrounded by a circle of hooklets, two of the four obtuse processes or suckers and the round caudal cyst behind. The average length of these as measured by a micrometer was one-eightieth of an inch. In others again of which we had apparently an anterior view, the entire circle of hooklets were clearly discerned; in these the obtuse processes were invisible. Some of the animals represented in the plate seem to be in a less advanced state of development. A number of them of various forms were collected within thin pellucid vesicles or cysts, which being ruptured allowed of the escape of the animalcules, and a multitude of minute rounded particles immiscible in the surrounding fluid. During the examination I observed in the field of the microscope several detached spines which were sharp-pointed and slightly incurvated. Nothing was observed capable of throwing light on the mode in which these animalcules are developed. The containing hydatid is not propagated like the *acephalocyst* of man, in which the gemmule is detached from the interior of the cyst, but the young cyst is excluded from the external surface. In some of the larger specimens two or three young cysts of the size of currants were observed in progress of development between the layers of the parent cyst.

I have not been able to meet with any account of a case in which this rare and curious hydatid has been noticed in this country. In the Hunterian Collection, there is a preparation of the *Echinococcus Hominis* described in the printed catalogue as 'Hydatids, on the inside of which are small ones; human: two preparations;' but on inquiry of Professor Owen I find that there is no further account of it. The Echinococcus Hominis has been observed in only a very few instances on the continent, and neither Rudolphi nor Bremser had met with it. A well-authenticated example of its occurrence in the human brain is published by Rendtorf in a Thesis on Hydatids. The account given of the animalcules discovered in that case is very imperfect and in the plate in which they are represented, but by no means well, only the coronet of hooklets is figured; the obtuse processes or suctorious mouths are not apparent. Müller has more recently described the case of a young man treated by Professor Necker for renal disease who voided with his urine a large number of these peculiar hydatid cysts. His description of the animalcules within them is minute, and accords very closely with this account which I have given of them, as observed in this case. He remarks however that the vermiculi were not present in all the hydatid cysts, but that the cysts which contained them were exactly similar to those which were devoid of them. In the case which I have here related the animalcules were detected in all the cysts examined in the microscope." 389.

Mr. Curling has found in the Medical Gazette (vol. xiii. p. 207,) a brief notice of a case of abscess in the liver, discharging echinococci through an opening in the parietes of the abdomen, by Mr. Rose of Swaffham, Norfolk.

### VIII. Observations on the Mode of Union of Fractures of Flat Bones. By R. H. Meade, Esq. Lecturer on Materia Medica at the Middlesex Hospital.

Mr. Meade observes, that the experiments to determine the mode of union of fractures have been made on the cylindrical bones. It has been stated that, in fractures of the bones of the skull, and also of the other flat, and of the spongy bones, union is effected without the formation of any external or provisional callus; but he can shew that this statement is not generally correct.

He has made several experiments on the scapula, which is easily fractured, and is essentially a flat bone; the two tables of which it is composed being in contact, in a great part of their extent: it contains however a considerable quantity of spongy tissue in the neck. The mode of union has been carefully observed in fractures traversing both these parts of the bone.

He relates the particulars of nine experiments, which we need not detail. From these he concludes it may be deduced, that union is accomplished in the thick part of this bone, exactly in a similar manner as it is in the cylindrical bones; viz. blood is first effused into the different tissues surrounding the fractured part; this blood is next absorbed, and coagulated lymph deposited in the substance of the muscles, and in the neighbouring cellular tissue, so as to form them into a solid gelatinous mass. The periosteum which has been ruptured, is separated from the fractured edges, and becomes inflamed and thickened: lymph, which is usually of a redder colour than that which forms the external callus, is also effused between the fragments themselves. At a later period, the external mass decreases in size, the muscles return to their natural texture, and a firm layer of cartilaginous matter surrounds the fractured spot, with which the periosteum is blended. This callus adheres firmly to the surfaces of the bone and dips down between the fragments, the edges of which become rounded off by the absorbents. Ossification then takes place by the deposition of earthy particles in the cartilaginous matter.

The process by which union is effected in fractures of the flat part of the scapula differs in some respects from the preceding, and also varies in different cases, in consequence of some varieties in the mode in which the fracture has taken place. In those cases in which the bone has been completely broken through, with the periosteum covering it, as in experiments six and seven, very little injury seems to be occasioned to the surrounding soft parts; in consequence of the bone breaking very readily, from its thinness, and the fragments suffering but little displacement, and therefore giving rise to very little inflammation or deposition of lymph in the muscles and cellular tissue, except in the immediate proximity of the broken edges. A considerable quantity of callus, however, is deposited along the line of fracture, with which the periosteum is blended, as in the fractures of other bones, and this callus seems to become bony before any solid union is effected between the edges of the bone itself.

After some remarks of rather a conjectural character upon the periosteum, Mr. Meade goes on to observe—

"I have stated that the process of union of fractures of the flat part of the scapula varies in certain cases. I will now endeavour to point out these particular instances. In many experiments which I have performed, I have found that union is accomplished without the formation of any provisional callus. In the greater number of these cases the periosteum had remained entire, as in the fourth experiment which I have related; and I am inclined to suppose, that this circumstance will partly account for the absence of callus. In the first place, the fractured edges being prevented by the entire state of the membrane from irritating the muscles and surrounding textures, the inflammation which gives rise to the effusion of lymph and consolidation of these textures, so as to form the external capsule, is not produced: and the preliminary steps in the formation of the provisional callus are wanting. In the second place, however, why is it that little or no exudation takes place from the surface of the bone beneath the periosteum, so as to form a ridge under this membrane? The only explanation which I can find for this is, that the periosteum is very little separated from the surface of the bone in these cases, and lymph seems only to be effused where the connexion between these parts is destroyed. I find it mentioned by writers, that cylindrical bones are sometimes broken without the periosteum giving way. Mr. Gulliver refers to a specimen preserved in the museum of the King's College, London, where both bones of the fore-arm of a child were fractured, without the periosteum being injured. He does not say, however, whether any callus was here formed. I can scarcely conceive it possible that the cylindrical bone of an adult can be broken without the periosteum giving way at the same time, at any rate, on one side. In a few cases, which I have noticed, where no provisional callus had been formed, the periosteum had apparently given way, and here the only reason that can be assigned for the absence of callus is, that the broken fragments had remained accurately in contact, and the direction of the fracture had been such, that complete immobility had been preserved; under which circumstances it has been premised, that union might take place, simply by the deposition of ossific matter between the extremities of the fragments. It has been said, that where union is accomplished without the formation of provisional callus, the process is very slow; but in the eighth experiment which I have related, the upper edges of the fracture which had remained in contact, and which were covered by periosteum, were united by new matter effused between the fractured margins, as early as the thirteenth day; which new matter was of a cartilaginous consistence, and insensibly blended with the broken edges, and would doubtless soon have become osseous." 403.

Mr. Meade concludes with a notice of one or two other facts.

"It has been stated by Macdonald, and repeated by other authors, that the cartilaginous matter forming callus, differs from true cartilage by becoming tinged red, when the animal has been fed on madder. I have distinctly observed that this is incorrect in many cases; the bones of the body generally having been found coloured, as well as the new bony particles deposited in the callus; while the cartilaginous matter surrounding these fragments has been perfectly white. I have been, by this means, enabled to observe, that the new bony particles are deposited irregularly through the provisional callus, and do not first arise from the surface of the old bone.

The lymph effused from the edges of the fractured bones themselves, and which fills up the interval between them, differs from that forming the provisional callus, in having a peculiar red granulated appearance. Mr. Howship, and latterly Mr. B. Cooper, consider that the coagulated blood plugging up the ends of the bones, actually becomes organized; but I could not find any other point of resemblance between this new effused matter and coagulated blood, besides the reddish colour." 404.

So that the process of union in fractures of the scapula differs little from that of the common cylindrical bones.

### IX. Case of Aneurysm of the Arteria Innominata, in which the Carotid and Subclavian Arteries were tied. By W. Wickham, Esq. Surgeon to the Winchester Hospital.

Richard Colt, aged 55, admitted into the Winchester County Hospital, Sept. 17, 1839. He had been a sailor, and resided nine years in a tropical climate. Six months ago he had observed a small swelling, about the size of a hazel nut, situated just above the right clavicle, at about its middle; it was unaccompanied by any pulsation or pain, and it disappeared in about eight days; from which time, until about four weeks previous to the date of his entry at the hospital, he had no return of swelling, when suddenly his attention was attracted to another tumor about the same size, which presented itself just above the sternal end of the clavicle: this soon became painful, and the pain was much increased when he was in the recumbent posture. The pulsation too was now soon evident, and as the swelling enlarged it occasioned some difficulty in breathing; at the end of the four weeks he showed the swelling to Mr. Adams, a surgeon at Lymington, who considered it aneurysm, and advised his coming to the hospital.

On his admission, the swelling had attained to the size of a hen's egg externally; it seemed that the tumor extended over the carotid artery at its lower part, reaching as high as the transit of the omo-hyoideus muscle: it inclined somewhat also towards the subclavian artery: it had all the characteristics of aneurysm, and that of the innominata. The health of the man appeared tolerably good, with the exception of some degree of constitutional disturbance, arising from continued pain and difficulty of respiration.

On the 24th of September, the case was submitted to Sir Astley Cooper, who was on that day at Winchester. His opinion confirmed that already entertained, that the disease was innominatal, and his sanction was given to the experiment of tying the carotid and subclavian arteries.

Sept. 25th.—A ligature was placed on the carotid artery immediately above the omo-hyoideus muscle, which was somewhat pushed upwards by the tumor. The operation was completed without any difficulty or any unusual circumstance attending it. The arrest of the circulation through the vessel was complete. The immediate effect of the operation in no degree diminished or disturbed the functions of the brain. The aneurysmal sac evidently lessened as soon as the ligature was tied, but the pulsation continued, though certainly with less force. The trachea was almost immediately relieved from pressure by the reduction of the tumor, and thereby the troublesome cough and dyspnœa considerably lessened. The patient throughout the day well, and feeling much benefitted by the cessation of those distressing sensations which the pressure of the aneurysm had previously occasioned.

No unfavourable symptoms followed the operation. The ligature came away on the 14th day, after which time the patient was allowed to walk about, and at the end of three weeks he left the hospital contrary to advice, but under the pretext of having affairs to settle at home, and with the promise of returning at the end of a week or ten days. At this time the tumor appeared of the size to which it was reduced immediately after the operation, and the pulsation as before the carotid was tied.

The patient was now surrendered to the care of Mr. Adams, who watched the case, and urged the performance of the second operation. This, however, the patient refused until the 27th November, two months after the first. At this time his appearance was very wretched, the difficulty of breathing extreme, cough very frequent, and deglutition much impeded. The tumor had increased to more than double its original size, and especially it had extended outwardly so as to overhang nearly half the clavicle.

A consultation was held on the 2nd December, and, on the 3d, the operation was performed.

The patient had passed a night of great suffering, and more than ever from the difficulty of breathing, which continued to the time of the operation. When brought into the operating theatre, he was quite livid from the arrest of the circulation through the lungs, and his pulse excessively weak. He appeared to be almost at his last gasp from suffocation; and great fears were entertained lest he should expire under the operation. It was however agreed, that this was the only chance of relief, and inasmuch as the tumor had so decidedly lessened after the former operation, it was hoped that a similar effect might be produced by tying the subclavian. The operation was therefore undertaken without further delay in the following manner:—

The patient was laid on a table with his head and shoulders raised towards the light, so that it might fall from the skylight into the hollow of the incision. The skin being drawn down, an incision was made through the integuments upon the clavicle; it commenced near the acromion, and extended along the clavicle to the tumour, which now occupied about one third of the clavicular region : the incision terminated by being carried a little upwards by the side of the external jugular vein, which was distinctly visible, and distended in consequence of the difficult respiration. It divided the skin and platysma myoides ; the cervical fascia, now becoming exposed, by careful touches of the scalpel, and the aid of a director, was easily divided. The loose cellular tissue having been next cleared away, the situation of the artery was readily detected in its passage over the first rib ; but it lay so far beneath the tumour and clavicle, that some difficulty was experienced in this stage of the operation. At first one of the cervical nerves, which received a pulsation from its contact with the artery, was mistaken for the subclavian, and a ligature passed under it : this mistake being at once discovered, it was not tied, but drawn away by this means from the artery, so as to bring it into view. A ligature was then passed around the vessel, by means of an aneurysmal needle, made by Weiss, having an eye at the end of a spring which slips along a canula inserted into a firm handle (this needle being admirably adapted for the purpose). The artery having been firmly tied, the pulsation at the wrist ceased, the wound was dressed, and the patient put to bed. Relief from the dyspnœa was immediate, so much so that the man was able to walk with ease to his ward, and from that time he continued free from any inconvenient pressure on the trachea until he died, the direction of the growth of the tumour having been subsequently diverted *outwards* towards the right shoulder.

On the next day, the patient was in every respect well : the tumour had manifestly decreased, but pulsation in the sac continued, though less in force, as on the former occasion. No pulse to be felt at the wrist. The heat of the arm greater than the opposite. He was treated as under the previous operation. No unusual symptom occurred until Saturday evening, Dec. 7th, when he was suddenly seized with delirium and muttering, a considerable increase of the aneurysmal tumour, and violent pulsation of the heart and *left* carotid : it was so great as to shake the whole frame, and actually raise the head from its pillow. He was immediately bled to twelve ounces and took thirty drops of laudanum. The first part of the night was passed with but little diminution of the symptoms : after which however they gradually diminished, and by the following evening he became quite tranquil. From the time of this attack the tumour never diminished; on the contrary it gradually, though slowly, increased. His health continued to improve, and with the exception of occasional pain from distention of the swelling and pressure on the nerves over which the tumour was situated, his sufferings were comparatively mild.

On the 23rd of Jan. he became suddenly faint and weak with loss of appetite, which lasted a few days ; and he again recovered sufficient strength to feel anxious to quit the hospital.

On the 25th of Dec. the ligature came away in the dressings, and the wound speedily healed. He now sat up and smoked his pipe, which was his habit, and, although gaining no ground, feeling some confidence as to his ultimate recovery, he persisted in his desire to quit the hospital, which he did on the 5th of February. He returned to Lymington.

On the 15th of February Mr. Adams was called to him on account of profuse bleeding, which occurred in the evening : this was arrested by plugging and clots, but on the following morning, Feb. 16th, he bled again, and died without an effort.

Thus a period of about four months and a fortnight passed between the first operation and the death of the patient.

*Post mortem appearances on inspection.*—The *heart* was large and loaded with fat.

The *pulmonary artery*, nearly twice the natural size.

The *aorta* extremely dilated from its origin in the left ventricle through the whole course of thorax; specks of osseous matter appeared in its coats.

The *superior cava* was also greatly enlarged.

The aneurysm had emanated from the *arteria innominata*, below its division into subclavian and carotid arteries. Nearly half of the innominata was occupied by the origin of the aneurysm. A ligature upon the remaining part of the innominata would not have left space between it and the arch of the aorta for the formation of a clot, or adhesive matter.

The *sternum* was slightly absorbed at its upper part.

The *clavicle* had undergone progressive absorption from the pressure of the aneurysm upon its inner and lower surfaces, and its articulation with the sternum had been destroyed, so that the clavicle became lifted upwards.

The *right* subclavian artery was obliterated from the clavicle to the first rib.

The *right carotid artery* was obliterated behind the tumour from just above the upper edge of the omo-hyoideus.

The *aneurysmal sac* reached from the arteria innominata to the upper part of the thyroid cartilage.

The sac had burst upon its left side although it projected most upon the right side.

Another case is thus added to those in which the subclavian and carotid arteries have been tied unsuccessfully for aneurysm nearer the heart. Nor can the result surprise us. When we consider on the one hand the very questionable probability of success from tying vessels beyond an aneurysmal tumour in an artery so near the heart as the innominata—and on the other hand, the likelihood that the aorta, if not the heart itself, is in an unsound state, we shall be prepared for failure, astonished at good fortune. Indeed it must form a subject of deliberation whether operations of this nature can be recommended. Every unsuccessful one materially impairs the arguments for its repetition.

In the case before us Mr. Wickham appears to have exhibited the skill and boldness for which he is deservedly celebrated.

### X. CASE OF TUMOUR IN THE PELVIS, IMPEDING PARTURITION. By J. C. W. LEVER, Esq., Assistant-Accoucher to Guy's Hospital Lying-in Charity.

On January 19th, 1840, at 7 A.M. Mr. N. was called to Mrs. Colston, aged 28, in labour with her fifth child. Her previous labours had been remarkably quick, indeed so rapid. that upon one or two occasions, the child was born before the arrival of the surgeon. When Mr. N. saw her, he found that labour-pains had commenced twelve hours previously, and on examination, he detected a tumour projecting into the vagina, impressing him with the idea that the rectum was full of fæces. The os uteri was felt above the tumour nearly dilated, and the head of the child presenting, her pains occurred at regular intervals, and were tolerably strong; he ordered her to take a dose of castor oil immediately. At 1, P.M., the oil had operated well, and on making examination, the tumour was found to be pushed lower down: an enema was now administered, which acted very speedily: introducing his finger into the rectum, Mr. N. found the tumor was situated between the rectum and vagina, and on further examination, it was felt to contain fluid, and to the left side, there seemed to be a firm body, impressing the examiner with the idea that it resembled in feel, the upper extremity of a fœtus.

"The pains," continues Mr. Lever, "were now very strong, and the patient had but slight intervals of ease. Mr. N. having requested my opinion of the case, I attended, and found the tumour as large as a fœtal head, occupying so much of the pelvic cavity, that the finger could with difficulty be passed between the tumour and the symphysis pubis, and on examining her rectum, the coccyx could not be passed; her pains were very violent and frequent. I advised the evacuation of the fluid contents of the tumour, thinking that if this were done, sufficient room would be obtained for the birth of the child, without diminishing the head. Having guarded a common lancet, I made an opening into the tumour, through the vagina, when upwards of a pint of an oily fluid immediately escaped, the sides of the tumour collapsed, the pains continued, the head rapidly advanced, and in two hours from the time of operation, she was delivered of a living male child, which was soon followed by the secundines. On placing the hand on the abdomen, after delivery, the uterus was found perfectly contracted, while to the left side the firm tumour which formed part of the contents of the sac could be felt. The evacuated fluid, when cold, greatly resembled dripping." 416.

On the 20th May, after her confinement, she complained of forcing pains, and Mr. N. again found the tumour between the vagina and the rectum, and extremely tense.

According to Dr. G. O. Rees' analysis, the substance contained much cholesterine. In a note, Dr. Merriman approves of the puncture of the tumour through the vagina. In two cases it had been opened through the rectum with a less successful result, and Dr. M. also approves of the lancet, rather than the trocar.

If we are not misinformed, this case was related to the London Medical Society by Mr. Newth (the "Mr. N." of Mr. Lever's report) and published in the Lancet at the time. We cannot help thinking that a more explicit reference to Mr. Newth would have been well.

